# Barriers for women aged 50 years and older to accessing health care in Germany

**DOI:** 10.25646/6067

**Published:** 2020-06-30

**Authors:** Laura Krause, Lorena Dini, Franziska Prütz

**Affiliations:** 1 Robert Koch Institute, Berlin Department of Epidemiology and Health Monitoring; 2 Charité – Universitätsmedizin Berlin Institute of General Practice

**Keywords:** ACCESS BARRIERS, HEALTH CARE, WOMEN, GERMANY, HEALTH MONITORING

## Abstract

For older and very old women in rural areas, the long distances involved and the limited availability of services can make accessing general practitioners and specialist physicians difficult. Based on data from the recent wave of the German Health Update (GEDA 2014/2015-EHIS), we have analysed the barriers to accessing health care for women in the age group 50 years and older in Germany. 21.0% of the women reported having had difficulties getting an appointment for an examination when it was needed during the twelve-month period before the interview. Fewer than 5% of the women reported distance as the reason for delayed medical examinations. Over the course of a one-year period, women in the large cities more frequently had to wait for an appointment for an examination than women in smaller towns. However, women in smaller towns reported more often that an examination had taken place late due to distance. The results are informative for the planning of health care services.

## Introduction

Towns and cities host a large proportion of public services [1]. The population density in rural regions is lower with fewer towns and large cities, and this frequently translates into issues with accessing services [1]. Access barriers to health care services in Germany have been described mainly for some regions in the former East German federal states [2, 3]. For example, while the majority of the population lives within a 20 minute drive to a primary care hospital [4], in some regions of Mecklenburg-Western Pomerania, Brandenburg and the north of Saxony-Anhalt this mark is exceeded [5]. The situation for outpatient care is similar [6]. In large cities, nearly 90% of the population lives within walking distance of a general practitioner (GP) or physician, compared to just over 60% in rural areas [6] – everybody else depends on having a car or access to public transport. However, more than one fifth of all German households did not have access to a car in 2017 [7], and this fact applies in particular to older and very old women primarily in rural regions [8]. Moreover, rural areas are not always well connected by public transport [9]. Low population density, great distances between homes and practices of GPs and specialist physicians, as well as limited public transport options are characteristic traits of many rural regions in Germany [10]. Older and very old women in rural areas therefore face difficulties in their daily routines, including doctor’s appointments [11].

Against this backdrop, we will now discuss the barriers to health care access (see Info box) women in the age group 50 years and older face in Germany. Data basis is the German Health Update (GEDA 2014/2015-EHIS) [12, 13], which allows us to draw conclusions on both geographic (greater distances) and organisational reasons (waiting time). The analysis was conducted within the project ‘Regionale Versorgung von Frauen über 49 Jahre durch Fachärztinnen und Fachärzte für Gynäkologie und für Allgemeinmedizin (Frauen 5.0)’ (‘regional availability of GP and gynaecological services for women aged 49 years and older, Frauen 5.0’) [14]. The project’s aim is, having described the current situation of care and having taken the perspectives of both patients and doctors into account, to develop future-oriented models capable of ensuring ambulatory gynaecological and GP care in particular for rural regions [14].

**Info box:**

**Hurdles to access health care services**

**The report of the European HealthACCESS project [15] describes six hurdles to health care access:**

**Table:** 

**Hurdle 1:**	The proportion of the population covered for health care
**Hurdle 2:**	Benefits covered by health care system
**Hurdle 3:**	Cost-sharing arrangements
**Hurdle 4:**	Geographical barriers to access (such as the remoteness of an area)
**Hurdle 5:**	Organisational barriers to access (such as waiting times)
**Hurdle 6:**	Utilisation of accessible services

## Indicator

GEDA is a nationwide interview survey of the adult population in Germany commissioned by the Federal Ministry of Health and conducted by the Robert Koch Institute (RKI) [12, 13]. The study is part of population-based health monitoring at the RKI, which aims to provide reliable data on population health, health behaviour and health care provision. Between 2009 and 2012, three waves of GEDA were conducted as telephone interview surveys, while GEDA 2014/2015-EHIS was based on a written questionnaire [12, 16]. The methodology of GEDA 2014/2015-EHIS is described in detail elsewhere [12, 13].

The GEDA 2014/2015-EHIS questionnaire included the question list of the European Health Interview Survey (EHIS) [12, 16]. The list is subdivided into four modules, whereby the second module on health care provision includes data on barriers to health care access. Participants were for example asked, whether they had experienced delay in health care in the past twelve months because the time needed to obtain an appointment had been too long (‘waiting time’). Then they were asked whether, in the past twelve months, they had experienced delay in getting health care due to distance or transport problems (greater distance) [16]. Respondents could answer ‘yes’, ‘no’ and ‘no need for health care’. Women who stated that they had not required treatment during the last twelve months were excluded from further analyses.

The first part of the analysis on waiting time is based on data from 5,532 women aged 50 years and older, the second part on greater distance, is based on data from 5,545 women aged 50 years and older. The results are presented as prevalences with 95% confidence intervals and stratified by age, socioeconomic status (SES) [17], place of residence [18, 19] and type of health insurance. Multivariate binary logistic regression was applied to determine whether the differences between groups are significant. The calculations were carried out applying a weighting factor that corrects deviations within the sample from the population structure (as at 31 December 2014) with regard to sex, age, type of municipality and education level. Type of municipality reflects the degree of urbanisation and corresponds to the regional distribution in Germany. The International Standard Classification of Education (ISCED) was used to classify the school and vocational degrees of participants [20]. A significant difference between groups is assumed if the calculated p-value is < 0.05.

## Results and discussion

21.0% of women aged 50 years and older had to wait for an appointment during the last twelve months (Table 1). The proportion of women who have to wait for an examination decreases with age: whereas around one third of 50- to 59-year-old women (29.1%) had to wait for an appointment during the last year, this figure drops to around one in seven women in the group 80 years and older (14.9%). Women in large cities (with a population of over 100,000 inhabitants) had to wait for an appointment for an examination more often than women in smaller towns with a population of up to 50,000 inhabitants. The proportion of women who waited for an appointment during the last twelve months is significantly higher among statutorily insured women compared to those covered by private insurance. No differences were found regarding SES (Table 1).

**GEDA 2014/2015-EHIS**

**Data holder:** Robert Koch Institute

**Aims:** To provide reliable information about the population’s health status, health-related behaviour and health care in Germany, with the possibility of a European comparison

**Method:** Questionnaires completed on paper or online

**Population:** People aged 18 years and above with permanent residency in Germany

**Sampling:** Registry office sample; randomly selected individuals from 301 communities in Germany were invited to participate

**Participants:** 24,016 people (13,144 women, 10,872 men)

**Response rate:** 26.9%

**Study period:** November 2014–July 2015

More information in German is available at www.geda-studie.de

For 4.9% of women aged 50 years and older, distance was the reason for having a medical examination late during the last twelve months (Table 1). This percentage varies with age and was highest for the oldest age group: whereas around one in twenty women in the 50- to 59-year-old group (5.4%) reported having had a medical examination late during the last year due to distance, it was about one in ten for women aged 80 years and older (10.4%). Compared to women with high SES, women with medium and low SES more frequently reported not having received a medical examination when they needed it during the last twelve months due to distance. Moreover, women living in smaller towns with fewer than 50,000 inhabitants more frequently reported not having received a medical examination when they needed it during the last year compared to women from large cities with ≥ 100,000 inhabitants. The proportion of women who reported having had medical examinations late is, moreover, higher for statutorily insured women than for those covered by private health insurance (Table 1).

Multivariate binary logistic regression analysis showed that the differences regarding age, SES, size of hometown and type of health insurance are statistically significant (data not shown).

GEDA 2014/2015-EHIS results indicate that fewer than 5% of women had a medical examination late due to distance. However, the results do show risk groups: women in smaller towns more often reported late appointments for an examination during the last twelve months due to distance than women in large cities. Furthermore, women aged 80 years and older and women with low SES more frequently reported having had medical examinations late due to distance than younger women and those with high SES. This is congruent with the fact that older and socially disadvantaged groups more often live in rural regions [1] with correspondingly greater distances to doctors [10]. However, the results of multivariate regression analyses show that age and SES have an impact on the accessibility of health services, even if the analyses are statistically controlled for place of residence and type of health insurance. In this context, it is also important to consider that health issues, such as loss of hearing, sight and the ability to walk are very common among very old women and women with low SES [21, 22]. A sensitivity analysis based on GEDA 2014/2015-EHIS data showed that women aged 50 years and older with health impairments more frequently report delay of medical examinations due to distance than non-health impaired women in this age group (8.8% versus 2.6%). For both of these two groups of women, therefore, a combination of various factors results in barriers to accessing health care services.

By contrast, the proportion of women who had to wait for an appointment for an examination during the last year was just over 20%. It is not possible to distinguish between the waiting times for GP and specialist physician appointments with the data available. Informative in this regard is a study by Germany’s National Association of Statutory Health Insurance Physicians (KBV). The more detailed analysis of outpatient care [23] revealed that patients get GP appointments more quickly than appointments with specialist physicians. This finding is also reflected in figures showing the level of satisfaction as regards accessibility to GPs and specialist physicians: whereas around one in seven people was not very satisfied with the access to specialists, it were only around one in 14 for GPs [24]. Nevertheless, waiting times in Germany for specialist physician appointments are among the lowest in international comparison [23, 25].

Compared to older age groups, 50- to 59-year-old women more frequently reported having had to wait for an appointment during the last year. According to the KBV study referenced above, urgent health issues and acute symptoms led to shorter waiting times [23]. As the risk of disease increases with age [21], it is likely that 50- to 59-year-old women do not have symptoms that require immediate medical examinations as often as old and very old women.

The analyses also revealed that statutory health insured women had to wait for an appointment for an examination far more often than those with private insurance. This finding is corroborated both by the study already referenced, as well as by a more recent KBV one, which both indicate longer waiting times for patients covered by statutory health insurance [23, 26]. Moreover, GEDA 2014/2015-EHIS data showed, that the proportion of women who had medical examinations late due to distance is also higher for women covered by statutory health insurance compared to the privately insured. This may be due to the fact that higher earners are more likely to be privately insured, and this group of people lives in the city more often than the general population [27]. In May 2019, Germany adopted the Appointment Service and Supply Act (TSVG), which aims to ensure that patients in statutory health insurance get an appointment with specialist physicians more quickly, by expanding the network of appointment service centres provided by the Associations of Statutory Health Insurance Physicians [28].

Long waiting times and limited geographical reachability can also indicate that there is a regionally disproportionate provision of medical services. According to GEDA 2014/2015-EHIS data, long waiting times appear to mainly affect women in large cities, whereas geographical barriers exist in particular for women in smaller towns. Analyses from Germany show that regional differences exist in particular with regard to specialist physicians [29–33]. It is very likely that demographic ageing will further exacerbate the impact of current issues on access to health care service [34]. This highlights the need for measures to ensure care which is capable of responding to actual needs. Besides adequately planning needs, instruments should include for example mobile practices or models of cross-sector care [35, 36]. A further Fact sheet in this issue of the Journal of Health Monitoring considers the likely development of the absolute numbers and proportions for older women in Berlin, Brandenburg and Mecklenburg-Western Pomerania.

## Key statements

- One in five women aged 50 years and older had difficulties getting an appointment for a medical examination or treatment when she needed it.
- One in twenty women aged 50 years and older reported delays to examinations or treatment due to distance.
- Women in large cities more frequently had to wait for an appointment for an examination than women in smaller towns. Due to distance, women in smaller towns often get appointments for examinations late.
- The proportion of women who report access barriers is higher for women in statutory than in private insurance.

## Figures and Tables

**Table 1:** Proportion of women aged 50 years and older who had to wait for an appointment for an examination (n =1,181) or who had an examination late due to distance (n=254) by age, socioeconomic status, place of residence and type of health insurance Source: GEDA 2014/2015-EHIS

Waited for an examination appointment			Examination late due to distance	
	%	(95% CI)	%	(95% CI)
**Total**	**21.0**	**(19.7–22.4)**	**4.9**	**(4.2–5.7)**
**Age group** 50–59 years 60–69 years 70–79 years ≥ 80 years	29.1 18.5 16.2 14.9	(26.8–31.5) (16.3–20.9) (13.9–18.8) (11.3–19.4)	5.4 3.4 3.6 10.4	(4.4–6.7) (2.5–4.6) (2.5–5.0) (7.4–14.5)
**Socioeconomic status** Low Medium High	21.6 20.9 19.7	(19.0–24.5) (19.3–22.5) (17.1–22.6)	8.3 4.2 1.7	(6.5–10.6) (3.4–5.1) (1.0–2.7)
**Place of residence** Municipality <50,000 inhabitants Municipality/town 50,000–< 100,000 inhabitants Large city ≥ 100,000 inhabitants	18.9 19.6 22.2	(16.5–21.5) (16.1–23.6) (20.6–23.9)	6.4 5.9 4.1	(5.0–8.1) (3.8–9.1) (3.4–5.1)
**Type of health insurance** Statutory insurance Private insurance	22.2 13.1	(20.8–23.8) (10.7–15.9)	5.2 2.3	(4.4–6.1) (1.5–3.5)

CI = confidence interval
